# Comparison of clinical pregnancy rates between hormone replacement therapy and modified natural cycle for endometrial preparation in frozen embryo transfer cycles: An RCT

**DOI:** 10.18502/ijrm.v23i5.19264

**Published:** 2025-07-29

**Authors:** Farnaz Safarloo, Marzieh Zamaniyan, Eisa Nazar, Keshvar Samadaee Gelehkolaee, Mahboubeh Omid, Sepideh Peivandi

**Affiliations:** ^1^Department of Obstetrics and Gynecology, Faculty of Medicine, Imam Khomeini Hospital, IVF Ward, Mazandaran University of Medical Sciences, Sari, Iran.; ^2^Department of Obstetrics and Gynecology, Faculty of Medicine, Sexual and Reproductive Health Research Center, Diabetes Research Center, Imam Khomeini Hospital, Mazandaran University of Medical Sciences, Sari, Iran.; ^3^Orthopedic Research Center, Imam Khomeini Hospital, Mazandaran University of Medical Sciences, Sari, Iran.; ^4^Department Of Reproductive Health and Midwifery, Faculty of Nursing and Midwifery, Imam Khomeini Hospital, Mazandaran University of Medical Sciences, Sari, Iran.; ^5^Department of Obstetrics and Gynecology, Faculty of Medicine, Sexual and Reproductive Health Research Center, Imam Khomeini Hospital, IVF Ward, Mazandaran University of Medical Sciences, Sari, Iran.

**Keywords:** Embryo transfer, Pregnancy rate, Vitrification, Hormone replacement therapy, Ovulation induction.

## Abstract

**Background:**

Frozen-thawed embryo transfer (FET) during the endometrial receptivity window is important for implantation.

**Objective:**

This study aims to compare the clinical pregnancy rate in 2 methods of endometrial preparation in FET using the hormone replacement cycle (HRC) and the modified natural cycle (mNC).

**Materials and Methods:**

In this randomized clinical trial, 128 infertile women who visited the Imam Khomeini hospital infertility clinic, Sari, Iran between April and October 2024 were randomly assigned to 2 groups (n = 64/each): the mNC frozen embryo transfer group and the HRC group. In the mNC frozen embryo transfer group, ovulation was induced using human chorionic gonadotropin, and the timing of frozen embryo transfer was scheduled based on ovulation. The HRC group received estradiol valerate until the endometrial thickness reached 8 mm, then daily injections of progesterone were added and FET were performed.

**Results:**

The participants had no significant differences in demographic characteristics. The primary outcome was clinical pregnancy rate with no significant difference between two groups (p = 0.282). No significant differences were observed between the mNC and HRC groups regarding the secondary outcome, which included human chorionic gonadotropin positive rate, chemical pregnancy rate, implantation rate, ongoing pregnancy rate, early miscarriage, ectopic pregnancy, twin pregnancy, and cycle cancellation rate. Significant differences were observed in the number of monitoring visits between the mNC frozen embryo transfer and HRC groups (p = 0.001).

**Conclusion:**

Although the results indicate that the impact of both methods is similar, the fact that fewer visits are required in a natural cycle and there is also less need for hormones could make it preferable.

## 1. Introduction 

Over the past 10 yr, the use of frozen-thawed embryo transfer (FET) has increased, driven by advances in assisted reproductive technology (ART). FET enables the transfer of a single selected embryo, lowers the risk of ovarian hyperstimulation syndrome, improves endometrial receptivity, allows for preimplantation genetic testing, and supports fertility preservation (1). Successful implantation requires a viable embryo, a receptive endometrium, and an optimal embryo transfer technique (1, 2). Although about 80% of in vitro fertilization (IVF) procedures progress to embryo transfer, only a small proportion result in pregnancy (2, 3).

The method of preparing the endometrium for embryo transfer significantly impacts IVF success rates (4, 5). As ovarian stimulation often produces more embryos than required for fresh transfer, freezing and transferring thawed embryos using advanced technologies improves the overall quality of infertility treatment (6). Frozen embryo replacement cycles account for approximately 25% of all ART-related births (7). This process is particularly important in ensuring optimal endometrial receptivity (8). Several FET protocols are available, and identifying the superior option remains challenging (9).

The 3 main protocols for endometrial synchronization in FET include natural cycles (NC-FET), modified natural cycles (mNC-FET) with human chorionic gonadotropin (HCG) trigger, and artificial replacement cycles. These protocols can be modified by adding progesterone for luteal phase support (LPS) in NC or mNC-FET cycles (10). In hormone replacement cycles (HRC), exogenous estrogen is administered to stimulate endometrial development while suppressing follicular growth. The HRC method offers greater flexibility and convenience, enabling better transfer scheduling and lowering the risk of cycle cancellation compared to natural cycles (11, 12). Currently, outcomes between these 2 methods differ; thus, despite conflicting data on their effectiveness, it remains uncertain which is superior. Therefore, this study was designed to evaluate and compare the success of frozen embryo transfer cycles using HRC and mNC methods of endometrial preparation.

## 2. Materials and Methods 

### Study design and participants 

The present study is a single-blind prospective randomized clinical trial (RCT) conducted from April to October 2024.

Sampling was conducted from April to August, and participants were followed up until October 2024. A total of 128 participants who met the inclusion criteria for FET were recruited, received the appropriate treatment plan, and were randomized into 2 groups of 64. Data from FET cycles in the original RCT were analyzed to compare the clinical pregnancy rate and pregnancy outcomes of 2 different endometrial preparation protocols in ovulatory women: 1) mNC-FET and 2) HRC. The study included 128 infertile women without ovulation disorders who underwent FET.

### Inclusion and exclusion criteria

The inclusion criteria were women aged > 20 and ≤ 40 yr, with a body mass index (BMI) between 18 and 30, regular menstrual cycles ranging from 21–35 days, undergoing their first or second FET cycle, and having at least one or 2 good-quality embryos. Women were excluded if they had diagnosed uterine anatomical abnormalities (congenital or acquired), hydrosalpinx, endometriosis, ovulation disorders, required preimplantation genetic testing, had a history of recurrent miscarriage or recurrent implantation failure, or had medical conditions contraindicating ART or pregnancy (13, 14).

### Sample size

According to a similar study (15) and using the following formula by considering an α error of 0.05 and a β error of 10% ${(z}_{0.975}=1.96,z_{0.90}=1.24,s_{1}=0.79,s_{2}=0.60,{\bar{x}}_{1}=8.01,{\bar{x}}_{2}=8.60)$ , the minimum required sample size in each group by assuming a 50% dropout rate was obtained from about 50 participants. Finally, to increase the accuracy and power of the study, 64 samples were collected in each group (a total of 128 participants). 


$$n=\frac{{\left(z_{1-\frac{\alpha }{2}}+z_{1-\beta }\right)}^{2}*\left(s_{1}^{2}+s_{2}^{2}\right)}{{\left({\bar{x}}_{1}-{\bar{x}}_{2}\right)}^{2}}$$

### Randomization

The participants were randomly divided into 2 groups, mNC-FET and HRC-FET, in a 1:1 ratio. A total of 128 eligible women were enrolled and randomly assigned: 64 participants to group A mNC-FET and 64 to group B HRC-FET. Randomization was carried out using a simple, alternate allocation method by the IVF unit secretary. In this method, the first eligible participant was assigned to group mNC-FET, the second to group HRC-FET, the third to group mNC-FET, and so on, until the target sample size was reached. It should be noted that, due to practical constraints, allocation concealment was not applied in this study.

### Blinding 

In the present study, the embryologist and data assessor were blindto treatment groups. Therefore, participants were not blinded to comply with ethical issues and were given the right to choose.

### Embryo cultured, embryo quality scoring system, vitrification, and warming

All participants underwent controlled ovarian stimulation following the antagonist protocol, with triggering using either gonadotropin releasing hormone agonist or HCG. Oocyte retrieval was performed 34–36 hr after triggering. Embryos were cultured at 37 C in an atmosphere containing 6% CO_2_. All embryos were graded prior to freezing. Cleavage-stage embryos were ranked based on morphological criteria, including size, number, uniformity, and the proportion of blastomeres.

Embryo quality on day 3 was assessed according to the Istanbul Consensus Workshop criteria (4). In this study, 3-day embryos of good quality were defined as having 6–10 cells and a grade 2 classification (10–25% fragmentation, predominantly uniform blastomeres, and no multinucleation). High-quality embryos were defined as those with 6–10 cells and a grade 1 classification ( < 10% fragmentation, evenly sized blastomeres, and no multinucleation). On day 5, embryo quality was evaluated using the Gardner and Schoolcraft scoring system, which considers the expansion grade (1–6), internal cell mass morphology (A-C), and trophectoderm morphology (A-C), based on the Istanbul Consensus Workshop criteria. Embryos with a score of ≥ 3 BB (AA, AB, BA, or BB) were considered of good quality. All embryos were frozen using the cryopreservation method on days 3–4 following microinjection. In brief, embryo freezing was performed using a vitrification kit (RS Medical, Iran). The criteria for successful embryo resuscitation after warming included survival of at least half of the blastomeres of the cleavage-stage embryo.

### The endometrial preparation protocols

The 2 employed protocols include the mNC-FET and HRC approaches. For all cases, on day 2 of the cycle, a basic ultrasound is performed, and if there is no follicle > 10 mm, it is planned to be placed in any of the endometrial preparation protocols.

#### Modified natural cycle frozen embryo transfer protocol

In the mNC-FET group, participants underwent transvaginal ultrasound examinations on the 2^nd^ and approximately the 12^th^ day of their menstrual cycle to assess the size of the dominant follicle and determine the optimal timing for ovulation triggering. If ovulation timing could not be confirmed on day 12, an additional transvaginal ultrasound was performed 2 days later. Ovulation was induced with an HCG injection (either 250 µg OvitrelleⓇ or 10,000 IU PregnylⓇ) once the dominant follicle measured ≥ 18 mm and the endometrial thickness (ET) was ≥ 8 mm. The cycle was canceled if no dominant follicle ( ≥ 16 mm) was observed by day 16.

LPS was provided with 400 mg of vaginal progesterone (Fertigest, Aburaihan Co., Tehran, Iran) administered twice daily, starting 48 hr after the HCG injection. The FET was scheduled based on the ovulation day, designated as day 0; cleavage-stage embryos were transferred on day 5, and blastocysts on day 7 following the ovulation trigger. In cases of confirmed clinical pregnancy, vaginal progesterone was continued until the 8^th^ wk of gestation. The number of visits from the beginning of the cycle to the day of embryo transfer was calculated based on the participant's medical records.

#### HRC frozen embryo transfer protocol

Women in the HRC group received estradiol valerate (2 mg tablets, Aburaihan Co., Tehran, Iran) twice daily, starting on the second day of their menstrual cycle. On the beginning of day 10 of the cycle, an infertility specialist performed regular transvaginal ultrasound scans to monitor ET. If the endometrial lining was deemed insufficient, the estrogen dose was increased. If, after 18 days of estrogen administration, the ET remained inadequate, the cycle was cancelled.

Once the ET reached 8 mm, participants were instructed to begin daily intramuscular injections of progesterone (100 mg). The day progesterone administration began was designated as P1. Cleavage-stage embryos were transferred on day P4, and blastocyst transfers were performed on days P5-P6.

**Figure 1:**
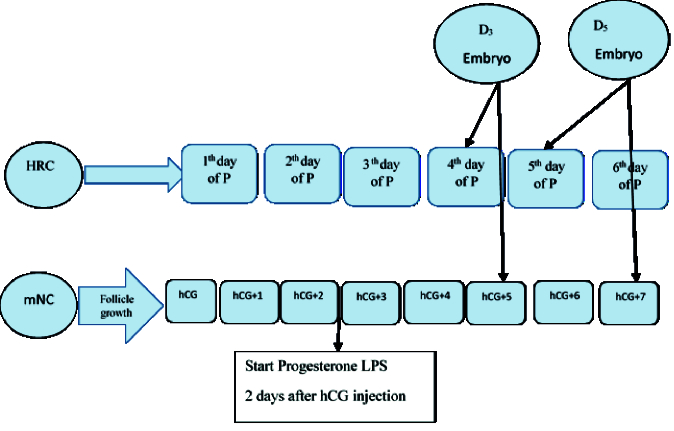
HRC protocol and embryo transfer timeline in the HRC group.

Both estradiol and progesterone treatments were continued for all participants for up to 2 wk following embryo transfer. In cases of confirmed clinical pregnancy, HRC was maintained until the 10 ${}^{\text{th}}$ wk of gestation (Figure 1). The number of clinic visits from the start of the cycle to the day of embryo transfer was recorded based on each participant's file. Intervention process (HRC, mNC, ET, progesterone, estradiol, LPS).

### Embryo transfer 

The decision to transfer one embryo or 2 embryos, was made by the physician, considering the participant's age, the number of previously transferred embryos, and individual factors associated with an increased risk of multiple pregnancies. Embryo transfer was performed under abdominal ultrasound guidance using a soft catheter. Based on the participant's condition and the physician's clinical judgment, either one or 2 cleavage-stage embryos, a single high-quality blastocyst, or one to 2 lower-quality blastocysts were transferred. Pregnancy was assessed by measuring serum β-hCG levels 14 days after embryo transfer. If the result was positive, β-hCG levels were monitored every 48 hr until reaching a minimum threshold of 1000 IU/mL. 2 wk later, a transvaginal ultrasound was conducted to determine the number of gestational sacs and assess fetal viability. LPS was provided to all participants for up to 2 wk following embryo transfer. In cases of confirmed chemical pregnancy, LPS was continued accordingly (16).

### Basic participation characteristics 

The baseline and clinical characteristics of the participants included age, BMI, anti-Mullein hormone levels, duration of infertility, number of transferred embryos, ET on the day of transfer, type and cause of infertility, history of previous pregnancy, ectopic pregnancy, abortion, and embryo transfer, as well as the embryo stage on the day of transfer and the ease of embryo transfer.

### Outcome measures

The primary outcome measured in this study was the clinical pregnancy rate, defined as the presence of a gestational sac and a fetal heartbeat detected via transvaginal ultrasound at the 8 ${}^{\text{th}}$ wk of pregnancy.

#### Secondary outcomes included

Positive hCG rate, defined as a β-hCG level > 50 IU/L was measured 14 days after embryo transfer, in accordance with laboratory guidelines. All positive results were followed until β-hCG levels reached a minimum of 1000 IU/mL. Biochemical pregnancy rate, referring to cases where a positive hCG test was recorded, but no clinical pregnancy was confirmed on transvaginal ultrasound at 8 wk. Early miscarriage, defined as the loss of a clinical pregnancy before the 12 ${}^{\text{th}}$ gestational week. Implantation rate, calculated as the number of gestational sacs observed via transvaginal ultrasound divided by the total number of frozen embryos transferred at the 6 ${}^{\text{th}}$ wk of pregnancy. Ectopic pregnancy rate, defined as the detection of a gestational sac outside the uterine cavity, is typically identified between the 6 ${}^{\text{th}}$ and 7 ${}^{\text{th}}$ wk of gestation. Twin pregnancy rate refers to the presence of 2 gestational sacs within the uterus at the 8 ${}^{\text{th}}$ gestational week. Ongoing pregnancy rate is defined as the continuation of pregnancy beyond the 12 ${}^{\text{th}}$ wk of gestation.

#### Cycle cancellation rate

In mNC-FET, cycles were cancelled if no dominant follicle ( ≥ 16 mm in diameter) was observed by day 16. In HRC protocols, cycles were cancelled if ET remained insufficient after 18 days of estrogen administration. Number of clinic visits, defined as the total number of visits from the start of the cycle to the embryo transfer. Notably, due to endogenous hormone production via the corpus luteum in mNC-FET cycles, no pharmacological LPS was required, distinguishing this protocol from HRC regimens.

### Ethical Considerations 

The study protocol was approved by the Ethics Committee of Mazandaran University of Medical Sciences, Sari, Iran (Code: IR.MAZUMS.IMAMHOSPITAL.REC.1402.125) and registered in the Iranian Registry of Clinical Trials (ID: IRCT20240302061147N1, updated on May 4, 2025). In addition, all participants were provided with comprehensive explanations regarding the endometrial preparation protocol by the attending physician, and informed consent was obtained prior to their participation in the study. The research was conducted at the Department of Obstetrics and Gynecology, School of Medicine, Imam Khomeini hospital, Mazandaran University of Medical Sciences, Sari, Iran. To ensure data protection from unauthorized access and disclosure, all participant information was collected and stored confidentially. No personal or identifying information was utilized during data analysis or reporting. The data were used exclusively for research purposes, and measures such as coding and secure storage were implemented to protect personal privacy and proprietary information.

### Statistical Analysis

Continuous and quantitative variables were summarized as mean ± standard deviation (SD), and categorical variables were expressed as frequency and percentage (%). The normality of data distribution was assessed using the Kolmogorov-Smirnov test. Depending on the distribution, independent samples *t* test or Mann-Whitney U test was applied to compare the means of normally and non-normally distributed quantitative variables between the mNC-FET and HRC groups, respectively. Associations between categorical variables were evaluated using the Chi-square test or Fisher's exact test, as appropriate. Further analysis was conducted using a multiple logistic regression model to identify factors associated with pregnancy outcomes among infertile women. Variables with a p < 0.25 in the univariate linear regression analysis were included in the multivariate model. All statistical analyses were performed using SPSS version 22 (SPSS Inc., Chicago, IL, USA), with a significance level set at p < 0.05.

## 3. Results

**Figure 2:**
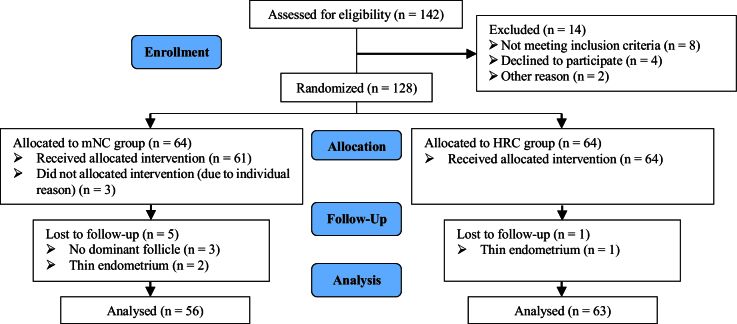
The schematic diagram for study design and data collection. mNC: Modified natural cycle, HRC: Hormone replacement cycle.

A total of 142 women treated at the IVF center of Imam Khomeini hospital Infertility Clinic, Sari, Iran were assessed for eligibility. 8 cases were excluded as they did not meet the inclusion criteria confirmed by the infertility fellowship. 4 women declined to participate, and 2 were excluded for other reasons. Ultimately, 128 participants were randomly assigned to 2 groups with 64 women in each group. In the mNC-FET group, 3 women did not receive the allocated intervention due to personal reasons, such as travel commitments or inability to take time off work. Consequently, 61 participants received the intervention in this group. During the course of the study, 5 cycles in this group were canceled due to the absence of dominant follicles or a thin endometrium. In the HRC-FET group, one cycle was canceled due to thin endometrium. No participants were missed to follow-up in either group. At the end of the study, 56 participants in group mNC-FET and 63 participants in group HRC-FET were included in the final analysis (Figure 2).

The Mann-Whitney U test revealed no statistically significant differences between the 2 groups in terms of mean age (p = 0.567), BMI (p = 0.691), AMH levels (p = 0.168), duration of infertility (p = 0.919), number of embryos transferred (p = 0.555), or ET (p = 0.117). Similarly, the Chi-square test showed no significant differences in the distribution of infertility type (p = 0.827), cause of infertility (p = 0.178), or other categorical variables between the groups. However, a significantly higher frequency of blastocyst-stage embryos was observed in the HRC group compared to the mNC-FET group (p = 0.017) (Table I).

**Figure 3:**
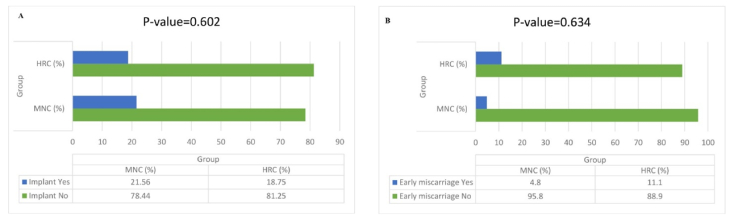
A) Comparison of implantation rate between the 2 study groups. B) Comparison of early miscarriage rate between the 2 study groups. Implantation rate: IR was defined as the number of gestational sacs determined by sonogram divided by the number of frozen embryos transferred, early miscarriage: Loss of clinical pregnancy before the 12 ${}^{\text{th}}$ gestational week. mNC: Modified natural cycle, HRC: Hormone replacement cycle.

As shown in table II, our Chi-square test analysis revealed no statistically significant differences between the mNC-FET and HRC groups in terms of the frequency distribution of chemical pregnancy rate (p = 0.640), implantation rate (p = 0.602), clinical pregnancy rate (p = 0.282), ongoing pregnancy rate (p = 0.221), early miscarriage rate (p = 0.634), ectopic pregnancy rate (p = 0.497), twin pregnancy rate (p = 0.999), and cycle cancellation rate (p = 0.109). However, the Mann-Whitney U test showed a significant difference in the mean number of monitoring visits between the mNC-FET and HRC groups (p = 0.001). Additionally, figure 3 illustrates the frequency distributions of implantation rate and early miscarriage, respectively, in both groups. As depicted, no significant differences were observed in early miscarriage (p = 0.634) and implantation rate (p = 0.602) between the mNC-FET and HRC groups based on the Chi-square test results.

To investigate the role of age in the association between the stage of embryo transfer and the occurrence of clinical pregnancy, we employed the Cochran-Mantel-Haenszel test, with the results presented in table III. Among women aged ≤ 35 yr and those > 35 yr, the relative risks were 0.76 and 0.45, respectively. This indicates that the likelihood of achieving a clinical pregnancy following cleavage-stage embryo transfer was 24% and 55% lower, respectively, compared to blastocyst-stage transfer in the 2 age groups. In other words, as age increased, the probability of clinical pregnancy following cleavage-stage transfer decreased relative to blastocyst-stage transfer. However, this difference was not statistically significant.

The factors associated with pregnancy outcomes were appraised as multiple logistic regression models (Table IV). Regarding biochemical pregnancy, the results of this model showed that no significant association was observed between any of the variables and the occurrence of biochemical pregnancy (p > 0.05). So, by adjusting the effect of other variables in the model, the odds of biochemical pregnancy in the HRC group were 1.04 compared to the mNC-FET group (p = 0.946). In other words, the odds of biochemical pregnancy in the HRC group were 4% more than mNC-FET group. Furthermore, regarding clinical pregnancy, a significant association was observed between the occurrence of clinical pregnancy with ET (p = 0.007) and embryo stage (p = 0.04). Therefore, by adjusting the effect of other variables in the model, the odds of clinical pregnancy in the cleavage stage were 0.36 times less compared to the blastocyst stage. Also, for each unit increase in ET, the odds of clinical pregnancy increase by 2.19 times. Besides, our results showed no statistically significant association between any of the variables and the occurrence of early miscarriage (p > 0.05). After adjusting for other variables, the odds of early miscarriage in the HRC group were 1.65 times higher than in the mNC-FET group (p = 0.718). Additionally, ET was significantly associated with ongoing pregnancy (p = 0.013). This suggests that for each unit increase in ET, the odds of ongoing pregnancy increase by 2.01 times. However, no significant associations were found between other variables and the occurrence of pregnancy outcomes.

**Table 1:** Comparison of baseline and clinical characteristics in HRC vs. mNC-FET endometrial preparation

**Variables**		**mNC-FET group (n = 61)**	**HRC group (n = 64)**	**P-value**
**Age (yr)^#^**		33.48 ± 5.54 35 (30–38)	33.16 ± 4.80 34 (29–37)	0.567
	**≤ 35^##^**	35 (57.4)	38 (59.4)	0.821
	**> 35**	26 (42.6)	26 (40.6)	
**BMI (Kg/m^2^)^#^**		27.08 ± 2.63 27.50 (25–29.90)	26.98 ± 3.36 28 (25.17–30)	0.691
**AMH (ng/ml)^#^**		3.48 ± 2.77 3 (2–4.10)	4.19 ± 3.19 4 (2–5.90)	0.168
**Length of infertility (yr)^#^**		4.89 ± 3.51 4 (2–7.50)	4.85 ± 3.64 4 (2–6.75)	0.919
**Type of infertility^##^**				
	**Primary**	36 (59)	39 (60)	0.827
	**Secondary**	25 (41)	25 (39.1)	
**Cause of infertility^##^**				
	**Male factor**	18 (29.5)	10 (15.6)	0.178
	**Female factor**	15 (24.6)	19 (29.7)	
	**Male and female factors**	28 (45.9)	35 (54.7)	
**History of previous pregnancy^##^**		25 (41)	28 (43.8)	0.754
**History of previous EP^##^**		5 (8.2)	6 (9.4)	0.816
**History of previous abortion^##^**		13 (21.3)	13 (20.3)	0.891
**History of previous transfer^##^**		21 (34.4)	23 (35.9)	0.911
**Embryo stage^##^**				
	**Blastocyst**	29 (51.8)	46 (73)	0.017*
	**Cleavage**	27 (48.2)	17 (27)	
**Ease of transfer^###^**				
	**Easy**	52 (92.9)	62 (98.4)	0.186
	**Difficult**	4 (7.1)	1 (1.6)	
**Number of transferred embryos^#^**		1.82 ± 0.38 2 (2–2)	1.78 ± 0.41 2 (2–2)	0.555
**Endometrial thickness (mm)^#^**		8.72 ± 0.80 8.50 (8–9)	8.53 ± 0.70 8.30 (8–9)	0.117
^#^Data are presented as Mean ± SD and MD (QR), Mann-Whitney U test. ^##^Data are presented as n (%), Chi-square test. ^###^Data are presented as n (%), Fisher's exact test. *Significant at the level of 0.05. mNC-FET: Modified natural cycle, HRC: Hormone replacement cycle, BMI: Body mass index, AMH: Anti-Mullerian hormone, EP: Ectopic pregnancy				

**Table 2:** Comparative evaluation of outcomes between the 2 methods of endometrial preparation using HRC and mNC

**Variables**	**Group**		**P-value**
	**mNC-FET group**	**HRC group**	
**HCG positive rate^#^**	25/56 (44.6)	27/63 (42.9)	0.845
**Biochemical pregnancy rate^#^**	4/56 (7.1)	6/63 (9.5)	0.640
**Implantation rate^#^**	22/102 (21.5)	21/112 (18.7)	0.602
**Clinical pregnancy rate^#^**	21/56 (37.5)	18/63 (28.6)	0.282
**Ongoing pregnancy rate^#^**	20/56 (35.7)	16 /63 (25.4)	0.221
**Ectopic pregnancy rate^#^**	0/56 (0)	2/63 (3.2)	0.497
**Twin pregnancy rate^#^**	1/56 (1.8)	2/63 (3.2)	0.999
**Early miscarriage rate^#^**	1/21 (4.8)	2/18 (11.1)	0.634
**Cycle cancellation rate^#^**	5/61 (8.2)	1/64 (1.6)	0.109
**Number of visit^##^**	2.80 ± 0.51 3 (2.25–3)	3.17 ± 0.58 3 (3–3)	0.001*
^#^Data presented as n (%), Chi-square test. ^##^Data presented as Mean ± SD and MD (IQR), Mann-Whitney U test. *Significant at the level of 0.05. HCG: Human chorionic gonadotropin, mNC: Modified natural cycle, HRC: Hormone replacement cycle, FET: frozen-thawed embryo transfer			

**Table 3:** Association between the stage of transferred embryo and clinical pregnancy by controlling the age groups

**Age (yr)**		**Clinical pregnancy**		**RR (95% CI)**	**P-value**
		**No**	**Yes**		
**≤ 35**					
	**Blastocyst**	23 (62.20)	14 (37.80)	0.76 (0.38, 1.52)	0.445
	**Cleavage**	22 (71.00)	9 (29.00)		
**> 35**					
	**Blastocyst**	24 (63.20)	14 (36.80)	0.45 (0.11, 1.71)	0.192
	**Cleavage**	10 (83.30)	2 (16.70)		
Data are presented as n (%), Cochran-Mantel-Haenszel test, RR: Relative risk					

**Table 4:** Analyzing factors associated with pregnancy outcomes using the multiple logistic regression

**Variables (References)**		**Biochemical pregnancy**		**Clinical pregnancy**		**Early miscarriage**		**Ongoing pregnancy**	
		**AOR (95% CI)**	**AOR (95% CI)**	**AOR (95% CI)**	**P-value**	**AOR (95% CI)**	**P-value**	**AOR (95% CI)**	**P-value**
**Group**									
	**mNC-FET**	-	-	-	-	-	-	-	-
	**HRC**	1.04 (0.26, 4.10)	0.946	0.61 (0.25, 1.45)	0.269	1.65 (0.10, 26.66)	0.718	0.66 (0.29, 1.53)	0.340
**BMI**		-		0.91 (0.73, 1.14)	0.269	1.65 (0.10, 26.66)	0.718	0.66 (0.29, 1.53)	0.340
**Endometrial thickness**		-		2.19 (1.24, 3.87)	0.007*	-		2.01 (1.15, 3.49)	0.013*
**Number of transferred embryos**		-		2.70 (0.83, 8.77)	0.098	-		1.62 (0.52, 5.01)	0.398
**AMH**		-		-		1.36 (0.95, 1.94)	0.081	-	
**Embryo stage**									
	**Blastocyst**	-	-	-	-	-	-	-	
	**Cleavage**	0.15 (0.01, 1.29)	0.086	0.36 (0.14, 0.95)	0.040^*^	-		-	
**Previous EP**									
	**No**	-		-	-	-		-	
	**Yes**			0.15 (0.01, 1.40)	0.098				
**Type of infertility**									
	**Primary**	-	-	-		-	-	-	
	**Secondary**	0.31 (0.06, 1.55)	0.155			2.26 (0.14, 35.78)	0.517		
**Previous abortion**									
	**No**	-		-		-		-	-
	**Yes**							0.90 (0.31, 2.62)	0.853
*Significant at the level of 0.05. AOR: Adjusted odds ratio, CI: Confidence interval, mNC-FET: Modified natural cycle, HRC: Hormone replacement cycle, BMI: Body mass index, AMH: Anti-Mullerian hormone, EP: Ectopic pregnancy									

## 4. Discussion

The present study aimed to evaluate and compare the outcomes of FET using 2 different endometrial preparation protocols: HRC and mNC-FET. The results demonstrated no statistically significant differences between the 2 groups regarding chemical pregnancy, implantation, clinical pregnancy, ongoing pregnancy, early miscarriage, ectopic pregnancy, twin pregnancy, or cycle cancellation rates. However, the mNC-FET group exhibited a higher clinical pregnancy rate compared to the HRC group (37.5% vs. 28.6%, p = 0.28). Additionally, the miscarriage rate was slightly higher in the HRC group compared to the mNC-FET group (11.1% vs. 4.8%, p = 0.58), though this difference was not statistically significant. This lack of significance may be attributed to the limited sample size and the study's insufficient statistical power. A post hoc power analysis based on the observed difference in clinical pregnancy rates revealed a power of approximately 19%, indicating that the study was underpowered to detect a statistically significant difference. Therefore, future research with larger sample sizes is warranted to better evaluate potential differences between these protocols.

It is important to consider that the absence of the corpus luteum in HRC-FET cycles may predispose women to complications such as preeclampsia and first-trimester pregnancy loss (1, 2, 13, 14, 16–20). The corpus luteum plays a vital role in early pregnancy by producing hormones such as estradiol and progesterone, essential for embryo implantation and pregnancy maintenance. It also secretes vasoactive substances like vascular endothelial growth factor and relaxin, which are crucial for early placental development. The absence of these factors in HRC cycles may adversely affect implantation and increase the risk of obstetric complications. Furthermore, accurate timing of embryo transfer in frozen-thawed cycles is critical for achieving favorable outcomes. Among the 2 preparation protocols evaluated, mNC-FET was associated with the highest clinical pregnancy rate, suggesting optimal synchronization between endometrial development and embryonic growth. In contrast, in HRC cycles, ET depends on administered estrogen levels. While higher estrogen doses can enhance ET, they may also negatively affect endometrial receptivity (13, 14, 16), potentially contributing to the slightly higher miscarriage rates observed in HRC cycles compared to natural cycles.

Carosso et al. investigated pregnancy outcomes across natural, modified natural, and artificial cycles. Their findings showed no significant differences in reproductive outcomes between natural/modified cycles and hormone therapy cycles. However, notably, pregnancies resulting from hormonally prepared cycles were associated with higher risks of gestational hypertension and abnormal placentation (17, 18). Similarly, Gale et al. concluded that timing FET in a true natural cycle yielded live birth rates comparable to those achieved with HRC protocols (19). In a study conducted by Rodrigues et al. in Brazil, the pregnancy rate was significantly higher in the mNC-FET group compared to the HRC group (20). Likewise, Moffa et al. in their analysis of 797 single-blastocyst frozen transfer cycles, reported no significant differences in pregnancy rates, clinical pregnancy rates, or miscarriage rates between HRC and mNC-FET protocols, which is consistent with the findings of the present study (21).

In 2022, a retrospective cohort study comparing true natural cycles, mNC, and AC was conducted. It was that the mNC protocol resulted in better outcomes, including a higher live birth rate compared to both NC and AC, while NC and AC were similarly effective (22). Similarly, in a randomized prospective trial, no significant differences in chemical, clinical, or ongoing pregnancy rates reported between mNC-FET and AC-FET groups (15). In contrast, Duzguner and colleagues showed that mNC-FET was associated with significantly higher biochemical pregnancy, clinical pregnancy, and LBRs (4). Huang et al. also found no significant differences in pregnancy outcomes between mNC-FET and hormone replacement therapy (HRT) cycles among women with regular menstruation (23). Likewise, Zhang et al. reported no notable differences between mNC-FET and HRT approaches (24). However, the current study observed a higher miscarriage rate in the HRT group compared to mNC-FET, highlighting the influence of endometrial preparation on outcomes (25, 26). Higher miscarriage rates in AC may result from hormonal imbalances, excess estrogen/progesterone, or thromboembolic risks, all of which can impair implantation and placentation. Additionally, differing outcomes may be due to non-ovulatory participants, such as those with polycystic ovarian syndrome or advanced maternal age (26, 27).

Furthermore, this study revealed a statistically significant difference in the average number of monitoring visits between the mNC-FET and HRC groups (2.8 ± 0.2 and 3.1 ± 0.8, respectively; p > 0.05). This finding suggests that ovulation induction with HCG may reduce the number of monitoring visits required to schedule frozen embryo transfer, potentially enhancing participant comfort and overall cost-effectiveness (1, 28). However, unlike HRC protocols, embryo transfer scheduling in mNC-FET protocols is less flexible in clinics that do not operate daily, presenting a logistical challenge. Additionally, only participants with regular ovulatory cycles are eligible for mNC-FET programs. HRC-FET protocols offer greater scheduling flexibility, allowing for better control of embryo warming and transfer timing. They also tend to reduce the rate of cycle cancellations compared to mNC-FET, largely due to ovulation suppression and the controlled administration of exogenous hormones (8). Nevertheless, our findings show no statistically significant difference in cancellation rates between the mNC-FET and HRC groups. These advantages of HRC-FET must be weighed against potential drawbacks, including a higher risk of thromboembolic events, hypertensive disorders in pregnancy, and increased financial costs related to the use of exogenous hormones (11, 12).

Although our results suggest that mNC-FET may offer benefits for participants with regular menstrual cycles -such as avoiding exogenous hormone exposure, reducing the risk of thromboembolic events, and possibly enhancing endometrial receptivity- HRT remains crucial for specific participant populations. HRT protocols are particularly advantageous for women with irregular or anovulatory cycles, including those with polycystic ovary syndrome or premature ovarian insufficiency, where endogenous ovulation cannot be reliably predicted. Moreover, the scheduling flexibility offered by HRT is essential for clinics with high participant volumes, international participants, or individuals facing occupational or logistical constraints. HRT may also be preferred in clinical scenarios such as recurrent implantation failure, where tighter hormonal regulation and a more predictable LPS are considered beneficial (11, 12). For women undergoing fertility preservation or those with contraindications to ovulation induction, HRT may represent the only viable option for endometrial preparation. In conclusion, while mNC-FET may be well-suited for selected participants with regular cycles, the choice of endometrial preparation protocol should be individualized, considering the participant's medical history, ovarian function, personal preferences, and logistical needs. Future prospective studies are needed to determine the most effective protocols for specific subgroups and to further personalize FET strategies (28).

### Strengths and limitations 

The strength of our study lies in its prospective design, which supports the use of a modified natural cycle for endometrial preparation in FET due to the presence of the corpus luteum. The corpus luteum produces key factors that contribute to uterine physiology and systemic maternal adaptation during early pregnancy. Furthermore, the study adjusted for major confounding variables, including age, BMI, and the cause of infertility. In addition, our findings suggest that HCG-triggered ovulation may enhance outcomes in conventional FET cycles. Among participants undergoing FET, ovulation stimulation was shown to significantly reduce the number of required monitoring visits for embryo transfer planning without compromising clinical outcomes. This approach may also improve patient comfort and the overall efficiency of the cycle. The primary limitation of this study is its single-center design, with a relatively small sample size and a limited study duration. Moreover, the study population included only ovulatory women and excluded individuals with ovulatory disorders. Future research should involve larger, multi-center prospective studies to confirm these findings and extend their applicability.

## 5. Conclusion

The results of our study indicate that no significant difference was observed in pregnancy outcomes between the mNC-FET group and the HRC group. In conclusion, mNC can be recommended for FET, particularly in participants with regular menstrual cycles, as they offer several advantages: greater physiological compatibility, increased convenience, reduced medication use, fewer monitoring visits, and clinical outcomes comparable to those of HRC.

##  Data Availability

The data sets generated for this study are available on request to the corresponding author.

##  Authors Contributions

F. Safarloo contributed to the design and conceptualization of the study, selection of methodologies, and drafting of the manuscript. M. Zamaniyan was involved in data collection, analysis, and interpretation of the results. E. Nazar was responsible for data analysis and interpretation of the findings. K. Samadaee Gelehkolaee assisted in determining the research methodology and editing the manuscript draft. M. Omid participated in data collection and manuscript writing. S. Peivandi played a key role in interpreting the results, editing the manuscript, and providing general feedback throughout all stages of the research.

##  Conflict of Interest 

The authors declare that the research was conducted in the absence of any commercial or financial relationships that could be construed as a potential conflict of interest.
